# The Relationship Between CDC Personnel Subjective Socioeconomic Status and Turnover Intention: A Combined Model of Moderation and Mediation

**DOI:** 10.3389/fpsyt.2022.908844

**Published:** 2022-06-22

**Authors:** Ying Shan, Guangwen Liu, Changqiang Zhou, Shixue Li

**Affiliations:** ^1^Centre for Health Management and Policy Research, School of Public Health, Cheeloo College of Medicine, Shandong University, Jinan, China; ^2^NHC Key Lab of Health Economics and Policy Research, Shandong University, Jinan, China

**Keywords:** subjective socioeconomic status, turnover intention, job burnout, grass-roots CDC personnel, expected salary change

## Abstract

A stable and motivated CDC workforce is critical for Chinese public health system improvement in the post-pandemic period of COVID-19. Meanwhile, the lack of career development prospects, low income, low status and the widespread and increasingly serious job burnout of employees CDC staff is a complex and difficult problem for the government. Therefore, this study explored the relationship between CDC personnel subjective socioeconomic status and turnover intention using a sample of 2,502 grass-roots CDC personnel who were administered with a subjective socioeconomic status scale, turnover intention scale, job burnout scale, and payment questionnaire. The results showed that: (1) subjective socioeconomic status had a significant association with job burnout and turnover intention; (2) all three dimensions of job burnout played a mediating role in the relationship between subjective socioeconomic status and turnover intention; (3) expected salary change played a moderating role between subjective socioeconomic status and turnover intention. The effect was stronger for workers with low expected salary change, which means due to the multidimensional comparative and complex mechanism of salary change, which had limited effect on turnover intention. These findings provide a basis for the relationship between turnover intention and socioeconomic status of grass-roots CDC personnel, and also provide ideas for reducing job burnout and staff turnover.

## Introduction

The COVID-19 pandemic has made more and more people to realize the importance of a robust public health system to respond appropriately to emerging and traditional health challenges (1, 2). Chinese center for disease control and prevention (CDC) personnel play a seminal role in epidemic prevention and control and promoting the health of the entire population; therefore, a stable personnel team is the foundation of China's public health system improvement (3). Unfortunately, due to the lack of career development prospects, low income and low status, the attrition of CDC personnel has been a serious problem in recent years. The number of CDC personnel in China was 206,485 in 2005 after the SARS pandemic but by 2020 was only 194,425. Therefore, exploration of the generation mechanism and influencing factors of turnover can not only mitigate the current situation of CDC personnel turnover, but also indirectly promote the reform of public health system.

Turnover has been a central topic for economists, psychologists, and management scholars (4, 5). Turnover intention reflects an individual's conscious and deliberate willfulness to quit one's job or organization within a certain period, which directly affecting the choice of departure (6, 7). A considerable number of predictive modeling formulas of voluntary turnover has been established, and researchers generally supported that the turnover intention is associated with social support, relationships, personal aspirations and family life (8, 9). Among them, personal will and choice are the fundamental factors, and career income and career prospect are the principal element that need to be considered.

The subjective socioeconomic status of CDC personnel refers to the individual's perception and identification of their position in the social and economic structure (10), and is strongly associated with a sense of social justice (11). Several studies suggested that subjective socioeconomic status reflects the individual's sense of belonging to the social class, and attitude toward future prospects, social phenomena and job choices (12, 13). According to the social comparison theory, team of employees do not work in a vacuum, they are always comparing and the results of that comparison will influence their career choice (14). In China, the huge income gap, low social status and poor career development path between CDC personnel and clinicians are important reasons for the loss of CDC personnel, especially at the grass-roots level, which are closely related to subjective economic status. Therefore, it is necessary to investigate the subjective socioeconomic status of grass-roots CDC personnel and its influence on turnover intention based on the actual situation of China.

In the late 1980's, Pines and Aronson defined job burnout as a state of physical, emotional and mental exhaustion (15, 16). The CDC personnel job burnout, refers to the emotional and behavioral exhaustion caused by the long hours and the lack of goals, which consists of three components referred to as emotional exhaustion, depersonalization and reduced personal accomplishment. In recent years, there has been a high incidence of burnout among medical staff, with a survey in 2010 showing that 52.4% of medical staff have job burnout (17). CDC personnel have higher levels of psychological stress and burnout compared with medical staff, 58.65% of grass-roots workers were detected job burnout in one research (18). Looking from the former researches, job burnout has a strong positive relationship with turnover intention (19). In addition to direct effects, we propose that job burnout serves as a mediator through which subjective socioeconomic status affects turnover intention as well (20).

Moreover, employees are often more focused on relative salary differences than absolute differences, and we called it expected salary change, which is the difference between expected income and actual income. A meta-analysis of 203 studies revealed that discrepancy between actual pay and deserved pay is the primary determinant of turnover intention and actual turnover (21). However, few studies have investigated the moderating effect of expected salary change between subjective socioeconomic status and CDC staff turnover intention and between job burnout. Therefore, it is necessary to examine the role of expected salary change in the mechanism of CDC personnel turnover intention generation.

To sum up, our study intends to select district and county-level CDC staff, a group with serious attrition problem, to investigate the impact of grass-roots CDC staff subjective socioeconomic status on turnover intention, the mediating role of job burnout between them, and the moderating role of expected salary change. This is of great significance for promoting the subjective socioeconomic status of grass-roots workers and decreasing their turnover intention.

### CDC Personnel Subjective Socioeconomic Status and Turnover Intention

In previous studies, scholars paid more attention to objective economic status, including social identity, occupational status, income level, education level and other factors, which can be used as a basis to divide individuals into higher and lower social classes (22). While, subjective socioeconomic status is reflected in how do individuals perceive and identify their position in the social and economic structure. CDC personnel subjective socioeconomic status refers to the position of CDC Personnel in a specific social class which is a comprehensive reflection of education, income, occupation and residential area (23). Scholars of various countries have been exploring the mechanism of confirming and evaluating the effect of socioeconomic status on individual physical and mental health from both subjective and objective aspects. However, there are not many research studies that directly study the relationship between subjective socioeconomic status and turnover intention.

Since the outbreak of COVID-19, the work content and intensity of grassroots CDC personnel have greatly increased, but their professional effort is severely disproportionate to the reward received. As a matter of fact, grass-roots CDC staff are facing serious self-development stress, such as poor salaries, limited development prospects and low social status, which will affect their cognition of subjective socioeconomic status results and have the intention to leave. Therefore, there is an urgent need to investigate the current situation of Chinese CDC staff subjective socioeconomic status and the possible impact on turnover intention.

Previous research studies revealed that people with different income levels and occupational status have significantly different turnover tendencies, and had a predictive effect on turnover intention. Based on these, we established our first hypothesis:

Hypothesis 1: Subjective socioeconomic status can significantly negatively predict turnover intention.

### The Mediating Role of Job Burnout

Job burnout refers to the emotional exhaustion caused by pressure from work or other reasons. According to current research, job burnout of CDC personnel is harmful in three aspects. The first is the impact on personal physical and mental health, such as depression and metabolic syndrome. Secondly, it will reduce work efficiency and cause tension in interpersonal relationship. Last but not least, long-term job burnout will have a huge impact on the organization of the individual, increase the turnover rate of CDC personnel, resulting in the instability of the CDC team (24). There is a stable negative correlation between job burnout and turnover intention. In addition, many studies in China have shown that the job burnout of CDC personnel is serious, for example, a survey of 244 CDC professionals in Sichuan province showed that the total detection rate of job burnout was 80.73%, and it may has increased in the COVID-19 pandemic.

There are not many research studies that directly study the relationship between subjective socioeconomic status and burnout. However, many studies have shown that education level, job title and income, which are important indicators of social and economic status, can have impact on job burnout. Subjective socioeconomic status is a comprehensive assessment of the subjective perception of an individual on the basis of a simple assessment of his or her social and economic status. We proposal that it is necessary to study the influence of CDC personnel subjective socioeconomic status on job burnout, and whether it will have an impact on turnover intention. Based on this, we established our second hypothesis:

Hypothesis 2: Job burnout plays a mediating role in subjective socioeconomic status and turnover Intention.

### The Moderating Role of Expected Salary Change

One of the key factors that determines turnover intention is job satisfaction, and the least satisfied in CDC staff of all is the salary (25). Pay satisfaction is largely determined by the discrepancies between actual salary and personal salary reference points, such as what employees feel they deserve, want, or see others receiving (26). When making job-related decisions, individuals simultaneously consider their salary bottom line, current pay level, and desired salary (27). As suggested by equity theory and the discrepancy models of pay satisfaction (28), we assume that A is in the clinical laboratory of CDC and B is in the clinical laboratory of hospital, however, in terms of work intensity: A=B; in terms of salary: A < B, whether individuals consciously or unconsciously compare their income levels horizontally or vertically. Horizontal comparison refers to whether labor compensation is directly proportional to work intensity under the same work intensity; vertical comparison refers to whether the salary will increase with the promotion of the position, mainly based on the establishment, professional title level and position of different expected income, that is expected salary change. Based on these, we established our third hypothesis:

Hypothesis 3: Expected salary change plays a moderating role in the direct path of the mediating path.

**Figure 1:**
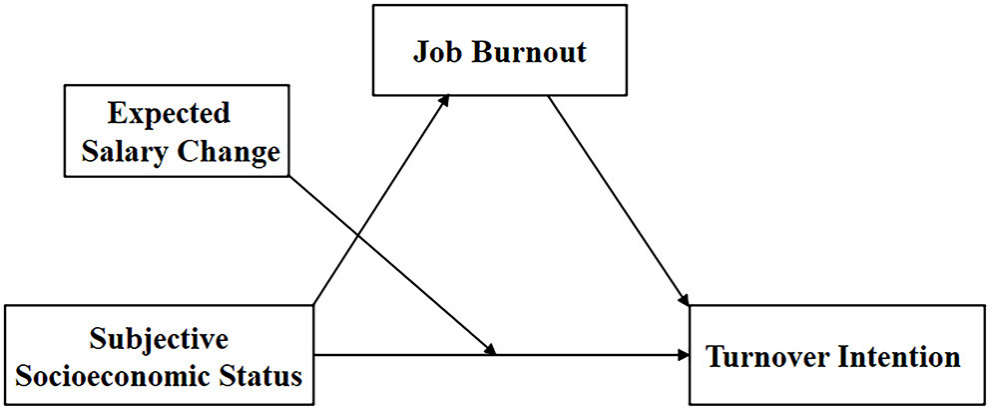
The proposed moderated mediation model.

In summary, this study constructed a model (as shown in Figure 1) to explore the mediating mechanisms of subjective socioeconomic status predicting the disease prevention and control personnel, to provide ideas for preventing and relieving the loss of CDC personnel. Three hypotheses were put forward: (1) subjective socioeconomic status has a significant predictive on turnover intention; (2) job burnout plays a mediating role in the relationship between SSS and turnover intention. (3) Expected salary change plays a moderating role in the direct path.

## Materials and Methods

### Participants

The participants included 2,502 county-level CDC staff from Shandong province, China. Stratified sampling was carried out according to the regional economic conditions, and 10 cities in Shandong province were selected to issue questionnaires in the form of electronic questionnaire. After excluding invalid questionnaires with incomplete answers, 2,502 valid questionnaires were obtained (valid recovery rate = 99.8%). Among the valid samples, there were 928 men (37.1%) and 1,574 women (62.9%). The average age of participants was 40.85 years (SD = 9.67), 2,043 (81.7%) were married, and 459 (18.3%) were unmarried; 864 (33.8%) had a college degree, 1,549 (61.9%) had a bachelor degree, and 107 (4.3%) had a master's degree; 249 (10.0%) Senior title, 928 (37.1%) had an intermediate title and 1,325 (53.0%) had a junior and below title. The years of working experience ranged from 1 to 43 years, including 936 (37.4%) with 1–9 years, 634 (25.3%) with 10–19 years, 627 (25.1%) with 20–29 years, and 305 (12.2%) with over and 30 years of working experience in CDC. The average number of years of working experience was 14.93 years (SD = 11.08).

### Measures

#### Chinese Version of the Subjective Socioeconomic Status Scale (CSSS)

CSSS is translated from the Mac Arthur Scale of Subjective Social Status for adults developed by Adler et al. (29). The Subjective Socioeconomic Status Scale contains two items, which are “please determine your family's economic position based on the socioeconomic development of your province” and “please compare your social and economic status with those around you.” And each item was divided into 10 levels and the participants are asked to choose which step they are on. From bottom to top, a score of 1 represents those who have the least money, the least education and the least decent jobs, while a score of 10 represents those who have the most money, the most education and the most decent jobs, with higher socioeconomic status as they go up. In this study, the Cronbach's alpha for this scale was 0.90.

#### Burnout Questionnaire (MBI-HSS)

Job burnout is a kind of long-term reaction caused by the individual's inability to cope with the constant pressure at work, which is manifested as emotional exhaustion, depersonalization and personal accomplishment (30). The job burnout scale adopts the Chinese version of the original scale (31), with 22 questions and 7 points of self-evaluation. The scoring method is 0–6, 0 means “never” and 6 means “very frequently.” Three subscales were used to measure the three dimensions of job burnout, including emotional exhaustion (nine questions), depersonalization (five questions), and personal accomplishment (eight questions). The three dimensions of the burnout questionnaire were scored separately. In terms of emotional exhaustion and dehumanization, the higher the score was, the stronger the burnout degree was. In terms of personal achievement, the higher the score, the less burnout. Emotional exhaustion score below 19 is mild, 19–26 is moderate, and above 26 is high. A dehumanization score of <6 is mild, 6–9 is moderate, and above 9 is high. Job satisfaction scores above 39 are mild, 34–39 is moderate, and below 34 is high. When all three aspects are high, it is considered that there is a high degree of job burnout. In this study, Cronbach's α of the total scale and three dimensions were 0.86 and 0.90, 0.81, and 0.87, respectively, showing high intrinsic reliability.

#### Turnover Intention Scale

The Turnover Intention Scale was developed by Tao et al. and has been widely used in sociological field (32). It consists of four items, each rated on a five-point Likert scale [1 = completely disagree to 5 = completely agree). Participants are asked to rate each statement, for example, “I will look for other job opportunities,” based on how much they agree or disagree with it. Higher scores indicate higher employee turnover intention. In this study, the Cronbach's alpha for this scale was 0.92.

#### Payment Questionnaire

The payment questionnaire is designed on the basis of literature review (33), including three questions: actual income, whether they are satisfied with the current income, and expected income. Expected salary change represents the difference between actual and expected wages (expected salary change = expected salary-actual salary).

### Data Analysis

SPSS version 25.0 (IBM, NY, United States) was used for the statistical analysis. Descriptive statistics were produced for all variables, while the PROCESS macro for SPSS (Model 4) was applied to examine the mediating effect of job burnout. Finally, the PROCESS macro for SPSS (Model 5) was used to examine the moderated mediating effect of expected salary change on the direct path (8).

## Results

### Common Method Deviation Test

As all the survey data were from the CDC staff self-reports, there may be common method deviation. Therefore, the Harman single factor test was used to test the deviation of variables. The results showed that the eigenvalues of 6 factors were >1, and the explanatory power of the first factor was <40% of the critical value (the value of variation was 31.99%). Therefore, common method bias did not affect the data results.

### Preliminary Analysis

The descriptive statistical results are shown in Table 1. The results showed that grass-roots CDC staff subjective socioeconomic status was negatively correlated with emotional exhaustion (*r* = −0.19, *p* < 0.001), depersonalization (*r* = −0.09, *p* < 0.001) and turnover intention (*r* = −0.21, *p* < 0.001), and was positively correlated with personal accomplishment. Emotional exhaustion and depersonalization were positively correlated with turnover intention (*r* = 0.53, *p* < 0.001; *r* = 0.37, *p* < 0.001), and personal accomplishment was negatively correlated with turnover intention (*r* = −0.13, *p* < 0.001). In addition, expected salary change was positively correlated with turnover intention (*r* =0.16, *p* < 0.001). A *t*-test was conducted to assess whether there were gender differences between the following variables. The results showed that there were significant gender differences in depersonalization, personal accomplishment, expected salary change and turnover intention (*t* = 4.06, *p* < 0.001; *t* = −4.68, *p* < 0.001; *t* = 4.51, *p* < 0.001; *t* = 6.35, *p* < 0.001). Compared with male CDC workers, female workers had higher burnout, expected salary change and turnover intention. The ANOVA on educational level shown that the higher degree workers had higher burnout and turnover intention.

**Table 1:** Descriptive statistics and correlation among variables (*N* = 2,502).

**Characteristics**	**1**	**2**	**3**	**4**	**5**	**6**	**7**	**8**	**9**
1. Gender	1								
2. Age	−0.16***	1							
3. Education level	0.09***	−0.33***	1						
4. SSS	0.03	0.07***	0.04*	1					
5. EE	−0.14	−0.16***	0.17***	−0.19***	1				
6. Depersonalization	−0.08***	−0.15***	0.05*	−0.09***	0.66***	1			
7. PA	0.10***	0.11***	−0.03	0.07**	−0.01	−0.14***	1		
8. ESC	−0.09***	0.04*	0.036	−0.18***	0.15***	0.10***	−0.03	1	
9. Turnover intention	−0.13***	0.05*	0.07**	−0.21***	0.53***	0.37***	−0.13***	0.16***	1
M	1.63	40.85	1.70	9.06	27.50	9.97	39.34	2,074.05	10.38
SD	0.48	9.67	0.54	2.94	12.45	6.13	10.86	2,326.48	3.70

*SSS, Subjective Socioeconomic Status; EE, Emotional exhaustion PA, Personal accomplishment; ESC, Expected salary change*.

^*^p < 0.05, ^**^p < 0.01, and

***p < 0.001.

### Mediating Effect Analysis

Model 4 of the PROCESS macro was used to investigate the predictive effect of CDC staff subjective socioeconomic status on turnover intention, and the mediating role of job burnout t (8). Since the scores of the three subscales of job burnout are independent of each other, the total score cannot be calculated together. Therefore, the effects of the three sub-dimensions on the subjective socio-economic impact on turnover intention were respectively analyzed by regression analysis. As Table 2 shows, subjective socioeconomic status was negatively associated with emotional exhaustion and depersonalization (β = −0.79, *t* = 9.69, *p* < 0.001; β = −0.16, *t* = −3.99, *p* < 0.001), which in turn was positively related to CDC staff turnover intention (β = 0.15, *t* = 29.49, *p* < 0.001; β = 0.21, *t* = 18.75, *p* < 0.001). And subjective socioeconomic status was positively associated with personal accomplishment (β = 0.21, *t* = 2.88, *p* < 0.001), which in turn was negatively related to CDC staff turnover intention (β = −0.33, *t* = −5.00, *p* < 0.001). In all three dimensions, the negative direct association between subjective socioeconomic status and turnover intention perpetration remained significant (EE: β = −0.14, *t* = −6.45, *p* < 0.001; DE: β = −0.22, *t* = −9.77, *p* < 0.001; PA: β = −0.2, *t* = −10.33, *p* < 0.001). Therefore, Hypothesis 1, 2 were supported. Job burnout partially mediated the relationship between subjective socioeconomic status and turnover intention [EE: indirect effect = −0.12, SE = 0.01, 95% CI = [−0.11, −0.07]; DE: indirect effect = −0.03, SE = 0.01, 95% CI = [−0.04, −0.01]; PA: indirect effect = −0.01, SE = 0.002, 95% CI = [−0.01, 0.000]]. The mediation effect of EE, DE, PA accounts for 46.54, 13.25, 2.71% of the total effect of subjective socioeconomic status on turnover intention.

**Table 2:** Fractional testing the mediation effect of subjective socioeconomic status on turnover intention (*N* = 2,502).

**Predictors**	**Model 1 (turnover intention)**		**Model 2 (emotional exhaustion)**		**Model 3 (turnover intention)**	
	**β**	***t***	**β**	***T***	**β**	***t***
Gender	−0.13***	−6.88	−0.99	−1.96	−0.99***	−6.82
Age	−0.01	−1.34	−0.13***	−4.95	0.01	1.36
Education level	0.53***	3.81	3.38***	7.19	0.02	0.18
SSS	−0.26***	−10.58	−0.79***	9.69	−0.14***	−6.45
Emotional exhaustion					0.15***	29.49
*R* ^2^	0.065	0.075	0.307
*F*	44.69***	52.05***	222.11***			
	**Model 1 (turnover intention)**		**Model 2 (depersonalization)**		**Model 3 (turnover intention)**	
	***β***	***t***	***β***	***T***	***β***	***t***
Gender	−1.03***	−6.88	−1.37***	−5.42	−0.75***	−5.28
Age	−0.01	−1.34	−0.10***	−7.48	0.01	1.36
Education level	0.53***	3.81	0.14	0.41	0.50***	3.84
SSS	−0.26***	−10.58	−0.16***	−3.99	−0.22***	−9.77
Depersonalization					0.21***	18.75
*R* ^2^	0.065	0.04	0.18
*F*	44.69***	26.31***	111.04***			
	**Model 1 (turnover intention)**		**Model 2 (PA)**		**Model 3 (turnover intention)**	
	***β***	***t***	***β***	***T***	***β***	***t***
Gender	−1.03***	−6.88	2.55***	5.66	−0.95***	−6.31
Age	−0.01	−1.34	0.12***	5.59	−0.01	−0.78
Education level	0.53***	3.81	−0.07	−0.17	0.53***	3.81
SSS	−0.26***	−10.58	0.21**	2.88	−0.25***	−10.33
PA					−0.33***	−5.00
*R* ^2^	0.07	0.03	0.07
*F*	44.69***	17.53***	41.09***

*SSS, Subjective Socioeconomic Status; PA, Personal accomplishment*.

^**^p < 0.01, and ^***^p < 0.001.

### Moderated Mediation Effect Analysis

**Figure 2:**
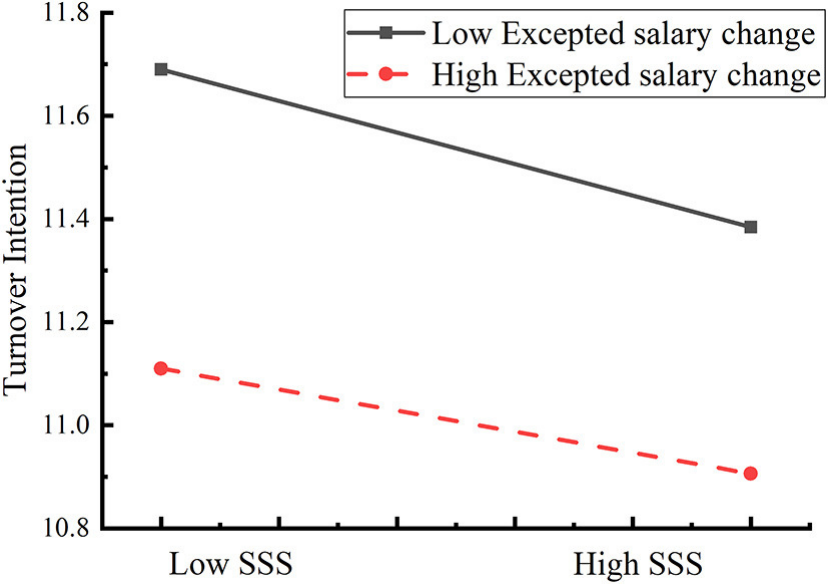
Interaction between expected salary change on job burnout.

To test the moderated mediation model, we used Model 5 of the SPSS PROCESS macro compiled by Hayes (8). After controlling for gender, age, and education level, the expected salary change moderation test was conducted; the results are shown in Table 3. As shown in the model (Turnover intention), the product of subjective socioeconomic status and expected salary change had a significant predictive effect on turnover intention (β = 0.05, *t* =2.01, *p* < 0.05). Therefore, we plotted predicted subjective socioeconomic status against turnover intention, separately for low- and high-levels of expected salary change (M ± 1SD). Simple slope tests showed that for CDC workers with high expected salary change, subjective socioeconomic status significantly predicted turnover intention, β_simple_ = −0.20, *t* = 11.69, *p* < 0.001. However, for CDC workers with low expected salary change, subjective socioeconomic status conflict significantly predicted turnover intention but to a much weaker extent, β_simple_ = −0.31, *t* = 11.11, *p* < 0.001 (Figure 2). The results showed that with the increase of expected salary change of CDC worker, the predictive effect of subjective socioeconomic status on turnover intention gradually increased.

**Table 3:** Testing the moderated mediation effect of subjective socioeconomic status on turnover intention.

**Predictors**	***R*** **^2^**	***F***	**β**	***t***
Model (turnover intention)	0.07	4.02*		
Gender			−0.13***	−6.86
Age			−0.03	−1.48
Educational level			0.08***	3.72
SSS			−0.20***	−10.44
ESC			0.07**	2.76
SSS*ESC			0.05*	2.01

*SSS, Subjective Socioeconomic Status; ESC, Expected Change*.

*p < 0.05, ^**^p < 0.01, and ^***^p < 0.001.

## Discussion

### The Effect of Subjective Socioeconomic Status on Turnover Intention

The results showed that subjective socioeconomic status had a significant negative predictive effect on turnover intention, so Research Hypothesis 1 was verified. It has confirmed a relationship between the subjective socioeconomic status and turnover intention of the Chinese grass-roots CDC staff, and expanded the application of social comparison theory in the Chinese context. At the same time, it is call on the government to pay attention to CDC personnel not only in the level of oral propaganda, but also to think about how to solve their practical difficulties, such as raising their salary, implement the epidemic prevention subsidy and optimize their career path (34). On the one hand, CDC personnel actively have carried out epidemic prevention and control work under the condition of low salary and low social recognition, which will lead to negative influence on their subjective social and economic identity. If measures are not taken to change the situation of CDC personnel, it is difficult to sustain the work of epidemic prevention and control under normal conditions by relying solely on spiritual motivation (35). On the other hand, CDC personnel also face real pressures in life, such as house, car, and their children education. If public health is not respected and the salary is not improved, many people will still leave the CDC after COVID-19 pandemic (36).

### The Mediating Role of Job Burnout

This study found that job burnout played a mediating role between the subjective socioeconomic status and turnover intention of grass-roots CDC personnel, so Research Hypothesis 2 was verified. COVID-19 pandemic has been going on for 2 years, and the long hours of high-intensity work and great mental pressure have placed a huge burden on the physical and mental health of CDC personnel. In the case of serious inequality between pay and income, it is easy to cause job burnout. To some extent, it fills a research gap by confirming the mediating mechanism between CDC staff subjective socioeconomic status and turnover intention. Low subjective socioeconomic status will aggravate job burnout, and then affect the turnover tendency of CDC personnel. Our study emphasizing the important role of personal subjective feelings in turnover intention, improving their compensation and treatment, and paying more attention to their mental health should become the important contents of the CDC system improvement, which is the core of solving the problem of staff turnover.

With the development of social economy, we are faced with more and more public health problems, and the work scope and pressure of disease control personnel are getting bigger and bigger (37). However, compared with clinical work, public health is not easy to see direct results, so it is not understood and valued by the public. Above all, clinicians and public health students are all medical school graduates, but CDC personnel earn half or even a third as much as doctors. Based on equity and social comparison theory, if low socioeconomic status is not addressed, it is likely to affect the emotional and psychological resources invested by CDC staff in their work, which leads to serious job burnout, and then produce turnover tendency (38).

### The Moderating Role of Expected Salary Change

The results of the moderating effect analysis showed that expected salary change played a significant moderating role in the path of subjective socioeconomic status to turnover intention, which verified Hypothesis 3. The results showed that the grass-roots CDC workers, who have higher expected salary change, will have higher degree of turnover intention (39). It may due to high expected salary change means the CDC staff were not satisfied with their actual income, which in turn lead to higher levels of job dissatisfaction and turnover intention. In addition, our study found that for CDC workers with lower expected salary change, although the degree of turnover intention was lower than workers with high expected salary change, the predictive effect of subjective socioeconomic status on turnover intention was stronger. Previous studies have shown that absolute compensation has limited impact on employee satisfaction, discrepancy between actual pay and deserved pay is the primary element. People with low expected salary change means, they are likely to be satisfied with their income and have stable cognition of subjective socioeconomic status, but this result is to the contrary (40). This may because expected salary change simply represents the longitudinal comparison of individual's income, that is, a comparison of their past, present and future. Subjective socioeconomic status includes not only horizontal comparison, but also vertical comparison, such as comparison with peer doctors and civil servants. More attention should be paid to relative compensation and the fairness of compensation between CDC personnel and medical staff, which is the ranger way to reduce staff turnover. In addition, individual with low expected salary change may pay more attention to social status, career development and other factors that are more difficult to change in a short time, thus affecting turnover intention.

### Practical Significance and Limitations

The current research has the following crucial theoretical and practical contributions. First, it is emphasized that more attention should be paid to the current situation of low social status and low salary of CDC personnel, which reflect that the problem of appreciating medical treatment but neglecting prevention is still outstanding. Second, during the fight against COVID-19, CDC staff are overworked, facing great work pressure and risk of infection, and the detection rate of job burnout is high. Relevant departments should provide appropriate care and support. The degree of job burnout can be alleviated by improving staff's self-identification with their professional value and social status and strengthening organizational support, and the talent team of disease prevention and control system should be expanded to relieve the working pressure of existing CDC staff. Finally, this study revealed that expected salary change has a complex role in subjective economic status, highlighting that raising wages alone will do little to reduce attrition, it should be considered from multiple dimensions such as fairness of salary distribution, career development prospect and social status.

Several limitations need to be considered when interpreting the findings. First, our cross-sectional data limit causal inferences. Previous studies have also indicated that job burnout can contribute to subjective economic status. Therefore, it is necessary to use longitudinal designs to obtain stronger empirical evidence of causal evidence in future research. This survey adopts the method of network non-probabilistic sampling, and the sample representativeness is limited to some extent. However, the conclusions and findings of this study still have certain reference value. Finally, although this study tested the moderating role of expected salary change between subjective economic status and turnover intention, future research needs to conduct a more in-depth study on more multi-level and multi-angle salary demand, to let CDC personnel get equal income with their efforts.

## Conclusion

In summary, this study is an important step forward in understanding how subjective socioeconomic status relates to the turnover intention of Chinese grass-roots CDC personnel. It has very important significance for the Chinese grass-roots CDC personnel who want to improve their socioeconomic status, and provides reference basis for the government to formulate corresponding measures to alleviate the loss of CDC personnel and reduce job burnout. First, it reveals the relationship between subjective socioeconomic status and burnout of grass-roots CDC personnel in the critical period of modernization improvement of disease prevention and control. Second, it shows that job burnout serves as a mediating role between subjective socioeconomic status and turnover intention, which highlights the important role of occupational mental health in socioeconomic status and talent force stability. Moreover, the relationship between subjective socioeconomic status and turnover intention is moderated by expected salary change, and the relationship appears to be stronger for CDC workers with low expected salary change than for those with high expected salary change. Finally, we hope that the government and society have more understanding and attention to the CDC personnel. Only understanding can bring understanding and empathy, and further bring participation, support, cooperation and construction.

## Data Availability Statement

The data that support the findings of this study are available from the corresponding author upon reasonable request.

## Ethics Statement

The studies involving human participants were reviewed and approved by Institutional Review Board of Shandong University. Written informed consent for participation was not required for this study in accordance with the national legislation and the institutional requirements.

## Author Contributions

SL was the principal investigator. YS and GL collected and analyzed the data under the supervision of SL. SL and YS designed the study, contributed to materials and analysis tools, and contributed to the writing of the manuscript. SL and CZ provided guidance to the manuscript. All authors contributed to the article and approved the submitted version.

## Conflict of Interest

The authors declare that the research was conducted in the absence of any commercial or financial relationships that could be construed as a potential conflict of interest.

## Publisher's Note

All claims expressed in this article are solely those of the authors and do not necessarily represent those of their affiliated organizations, or those of the publisher, the editors and the reviewers. Any product that may be evaluated in this article, or claim that may be made by its manufacturer, is not guaranteed or endorsed by the publisher.
